# The Effect of Gender Role Expectations, Sexism, and Rape Myth Acceptance on the Social Perception of Sexual Violence: A Meta-Analysis

**DOI:** 10.1177/15248380251343190

**Published:** 2025-06-21

**Authors:** Dominique Trottier, Valérie Laviolette, Irza Tuzi, Massil Benbouriche

**Affiliations:** 1 Université du Québec en Outaouais, Gatineau, QC, Canada; 2 Philippe-Pinel National Institute of Forensic Psychiatry, Montreal, Canada; 3 Univ. Lille, ULR 4072 - PSITEC - Psychologie : Interactions Temps Émotions Cognition, Lille, France

**Keywords:** sexual violence, gender role expectations, sexism, rape myth acceptance, meta-analysis

## Abstract

Recent systematic reviews have highlighted the role of certain attitudes in shaping the social perception of sexual violence. This meta-analysis aimed to assess the effect of sexism, gender-role expectations, and rape myth acceptance on the social perception of sexual violence. It also aims to determine whether attitudinal dimensions, participant gender, or the dyadic composition of sexual violence incidents moderate this relationship. A literature search covered published and unpublished manuscripts from January 1, 2010, to December 31, 2023, across PsychArticles, Proquest, and Google Scholar. Manuscripts had to measure at least one attitude and assess participants’ perception of material depicting sexual violence between two adults. Data from 40 studies from 10 different countries, involving 12,283 participants, revealed a significant association between attitudes and the social perception of sexual violence (*r* = .425, *p* < .001). Meta-regression highlighted the significant contributions of greater gender role expectations, hostile sexism, benevolent sexism, and rape myth acceptance to the social perception of sexual violence incidences. Their combined effect correlated with increased victim blame (*r* = .558), reduced victim credibility (*r* = −.492), decreased perception of incident severity (*r* = −.363) and victim trauma (*r* = −.390), heightened perceptions of victim pleasure (*r* = .417), as well as reduced perpetrator blame (*r* = −.288) and sanction severity (*r* = −.191). These tendencies were even more pronounced in cases involving same-gender perpetrators and victims. Participant gender was not a significant moderator. These findings underscore the need for prevention efforts to address broader gender biases and power dynamics.

## Introduction

There is a growing spotlight on the existence of a social climate tolerant of sexual violence. The World Health Organization (2022) defines sexual violence as “any sexual act, attempt to obtain a sexual act, or other act directed against a person’s sexuality using coercion, by any person regardless of their relationship to the victim, in any setting.” Research suggests that sexual violence tends to be more prevalent in cultures where social norms promote the objectification of women and foster gender inequity (Jewkes et al., 2015; Yodanis, 2004). In fact, culture significantly influences how populations and societies perceive, interpret, and respond to sexual violence (Kalra & Bhugra, 2013). The term *social perception* refers to the process by which individuals form impressions of themselves, situations, and other people (Fiske & Taylor, 2017). It involves interpreting social information and is, thereby, influenced by social context and culture. Over the years, the sociocultural perspective has emphasized the influence of attitudes and beliefs rooted in patriarchal values as factors contributing to the emergence and perpetuation of sexual violence (Murnen et al., 2002; Spencer et al., 2023; Trottier et al., 2019). These values are ingrained in power dynamics, gender hierarchy, and societal norms that perpetuate gender-based inequities.

### The Roots of Sexual Violence Tolerance

Patriarchal socialization is founded on two fundamental pillars: gender hierarchy and traditional gender roles. Within a patriarchal social structure, men are conventionally perceived as decision-makers or providers, while women are valued in supportive roles. Attitudes that convey the gender hierarchy—that is, the idea that men have a superior status to women—are commonly referred to as sexism. Over the years, the definition of sexism has evolved. Historically, the term sexism represented a hostile disposition toward women (Swim et al., 1995), which manifests through the expression of overtly dominant or contemptuous attitudes, a desire for control, or the normalization of men's use of violence against women (Glick & Fiske, 1996, 2001). As time progressed, the conceptualization of sexism expanded to include its hostile dimension as well as other forms of power imbalance, such as using a paternalistic or benevolent stance of decision-making, protecting, and providing for women (Glick & Fiske, 1996). Although referring to a chivalrous and romanticized vision of male–female relationships, the “benevolent dimension” of sexism relies on an unbalanced view of gender and reinforces power inequalities (Glick & Fiske, 1996, 2001). In fact, the conceptual basis underlying both the hostile and benevolent dimensions of sexism is the same: women’s social status is inferior to men’s, implying that it is men’s responsibility to either control or protect women (Glick & Fiske, 1996, 2001). This gender hierarchy is deeply entrenched within legislative, institutional, and social structures (Hunnicutt, 2009).

Socialization into patriarchal values not only establishes a gender hierarchy but also shapes cultural norms surrounding masculinity and femininity, generally referred to as gender role expectations. Gender role expectations refer to the idea that men and women are fundamentally different, possessing attributes, interests, and roles that are specific to their gender, mutually exclusive and complementary, and that the proper functioning of interpersonal and social relationships depends on the respect of these roles by both women and men. For example, socialization instills expectations that men exhibit assertive and aggressive traits, while women are expected to demonstrate compliance and passivity (Byers, 1996; Lips, 2018). In addition to their contribution to upholding the existing gender hierarchy, gender role expectations likely predispose us, as a society, to normalize men's aggression and transgressions.

### How Gender Role Expectations and Gender Hierarchy Shape the Social Understanding of Sexual Interactions

Both gender role expectations and gender hierarchy regulate the realm of sexuality. It introduces clear expectations about who men and women should be attracted to and how they should behave while interacting with each other. First, there is an assumption that men and women will be attracted to one another. This assumption, commonly referred to as heteronormativity, portrays heterosexuality as the universal, self-evident, and natural sexuality, which is endowed with a superior moral and greater value in reference to normative systems (Chamberland, 2019).

This viewpoint frequently results in the anticipation that individuals will engage in heterosexual relationships and adhere to specific behaviors associated with their gender (Chamberland, 2019). For instance, there is a gendered expectation that men want sex, are always ready for sexual intercourse, and will be the ones initiating physical contact. Conversely, it is expected that women should seek significant emotional investment from their partner and establish boundaries around their sexual interactions to delay the onset of sexual intimacy. A woman who explicitly or quickly expresses her desire for a sexual relationship, initiates physical contact, or offers little resistance to a man’s initial sexual advances would potentially face negative social reactions (Byers, 1996; Flood & Pease, 2009).

Consequently, it is expected that women will initially say no to men’s sexual advances (Muehlenhard & Rodgers, 1998). In reaction to a refusal, it is expected that men will persist in seeking intimacy and find ways to navigate the limitations imposed by women on a sexual level. Thus, the gender role expectations surrounding heterosexual sexual intimacy establish a heterosexual sexual script (Kim et al., 2007; Simon & Gagnon, 1986) that normalizes an element of persistence in men’s sexual advances toward women while attributing to women the responsibility of restraining men’s sexual behaviors.

### The Social Construction of Sexual Violence

Socialization into these gendered sexual norms influences the social construction of sexual violence, which is frequently limited to the concept of rape. Rape is often framed as the heterosexual sexual script taken too far, or in other words, as a sexual interaction in which a man exerts excessive dominance over a woman and overpowers her to achieve vaginal or anal penetration (Peterson & Muehlenhard, 2004). This narrow conception of sexual violence impacts the narratives surrounding the definition of sexual violence (Mortimer et al., 2019) and the way we perceive such instances. On the one hand, it establishes a hierarchy regarding the severity of sexual violence acts, thereby invalidating certain experiences that may not involve elements of physical overpowering or penetration by a penis, thus minimizing their severity or repercussions.

On the other hand, this perspective, deeply rooted in both heteronormativity and gender expectations, molds our societal understanding of who can be victims and perpetrators of sexual violence (Mortimer et al., 2019). There is an assumption that instances of sexual victimization are less severe for men, particularly when the perpetrator is a woman, or that men are rarely affected by sexual abuse (Davies & Rogers, 2006; Kia-Keating et al., 2005). Research has also emphasized the lack of visibility of female perpetrators and queer victims (Girshick, 2002; Stemple & Meyer, 2014). Women are traditionally viewed as physically weak and sexually passive, thereby abnegating the potential for female perpetrators of sexual violence. Additionally, within a heteronormative framework of sexual intimacy, women engaged in same-sex relationships are considered not to engage in “complete” sexual intercourse, which renders instances of sexual violence among sexual minority women inconceivable and invisible (Girshick, 2002).

### How Gender Role Expectations and Gender Hierarchy Shape Rape Myths

The interplay of gender hierarchy, gender role expectations, heteronormativity, sexual scripts, and representations of rape converge in a series of factually inaccurate but commonly held beliefs referred to as rape myths (Brownmiller, 1975; Burt, 1980). Several of these myths center around the victim’s behaviors at the time of the assault. Therefore, if a woman’s actions do not conform to gendered expectations of social or sexual behavior (e.g., if she has consumed too much alcohol, is dressed in a way that is perceived as provocative, or has initiated physical contact), she is considered to bear some responsibility for the assault she experienced (Grubb & Turner, 2012). Gendered expectations of men promoting dominance and physical strength generate myths suggesting that a “real man” could have fought back against his aggressor if he had truly wanted to, especially if the perpetrator was a woman. Conversely, behaviors of men who perpetrate sexual violence are also interpreted in the context of gendered expectations, allowing for their justification. A widely spread myth is that rape results from men not being able to control their sexual urges (Webster et al., 2018).

These myths are widely spread to this day in the general population (Edwards et al., 2011) and are endorsed by both men and women (Hockett et al., 2016). For example, Salmona (2019) lead a population-based study in France on representations of rape with 1,000 French citizens of varied genders and sexual orientations. Results revealed that a significant proportion of respondents believe that it is difficult for men to control their sexual desires (57%) and that a rapist’s responsibility is diminished if his victim behaved provocatively in public (42%). Baril et al. (2025) conducted a similar population-based study involving 1,222 Quebec (Canada) residents of varied genders and sexual orientations and found that the majority of respondents did not reject myths such as: “Men don’t usually intend to force sex on a woman, but sometimes they get sexually carried away” (72%) and “Women who say they were sexually assaulted sometimes gave men false hopes and later regretted it” (60%). These results also align with findings from Australia where 43% of a national sample of 8,731 men and women agreed that rape is the result of men not being able to control their need for sex (Webster et al., 2018).

In summary, patriarchal socialization, rooted in gender hierarchy and gender roles, establishes expectations and social norms surrounding sexuality. These norms, in turn, bias our social perception of sexual violence as well as how we perceive its victims and perpetrators.

### Rape Culture and Its Repercussions

The term “rape culture” is commonly used in reference to a social environment that condones sexual violence (Brownmiller, 1975). It recognizes the presence of social structures, norms, beliefs, and behaviors that contribute to trivializing sexual violence and shifting the responsibility onto the victims rather than on the perpetrators (Brownmiller, 1975; Burt, 1980; Murnen et al., 2002). Sexual violence trivialization can occur in several ways, including minimizing the severity of sexual violence incidents, normalizing unsolicited sexual attention or comments, denying the coercive nature of sexual behaviors, as well as negating the perpetrator’s intent. Such trivialization undermines the recognition of the physical, emotional, psychological, and economic consequences for those who experience it.

In addition to the trivialization of sexual violence, important aspects of rape culture are the attribution of blame to the victim and the questioning of victims’ credibility (Suarez & Gadalla, 2010). When victims report sexual violence incidents, it is often their decisions and behaviors at the time of the incident that are scrutinized rather than those of the perpetrator. The victim’s credibility can also be questioned by casting doubt on their ability to remember the events accurately, the reasons for their report, and the timing of their denunciation. This tendency to question the behavior and credibility of victims, rather than those of the perpetrator, revictimizes the victims, discourages them from reporting, and reinforces the idea that sexual violence prevention is the victim’s responsibility (Suarez & Gadalla, 2010). Moreover, a recent systematic literature review highlights greater negative social reactions for Black and Hispanic victims, less educated victims, as well as victims from sexual minorities (Ullman, 2023), further emphasizing the intersectional nature of the power inequities at play in sexual violence perception.

Several studies conducted over the years have employed diverse materials, such as vignettes, audio scenarios, videos, etc., to depict instances of sexual violence and assess participants’ perceptions of such incidents (Abbey & Wegner, 2015). Findings from these studies illustrate the relationship between greater endorsement of attitudes shaped by patriarchal socialization and a propensity to attribute greater blame and lesser credibility to victims, ascribe lesser blame and milder sanctions to perpetrators, display reduced recognition of the gravity of rape incidences or their impact on the victim, and entertain the notion that the victim derived pleasure from the incident (Ayala et al., 2018; McDaniel & Rodriguez, 2021).

In addition, several recent systematic reviews have highlighted that gender hierarchy, gender-role expectations, and rape myth acceptance all play a role in shaping social perceptions or reactions to sexual violence incidents (Hockett et al., 2016; Hyży, & Mitka, 2024; Murvartian et al., 2024; Trottier et al., 2023; Ullman, 2023). Although the relationship between these attitudes and the perception of sexual violence is widely acknowledged, it is crucial to examine the strength of these relationships in greater detail, to identify whether certain attitudes exert a stronger influence on the perception of sexual violence, and to determine which aspects of perception are most significantly affected by those attitudes. Such knowledge is essential both for understanding the underlying causes of the perception of sexual violence and for effectively guiding education and prevention strategies.

### Aim and Objectives

Through this meta-analysis, our primary objective is to assess the robustness of the relationship between attitudes shaped by patriarchal socialization, namely sexism (gender hierarchy), gender-role expectations, and rape myth acceptance and the social perception^1^ of sexual violence incidences. Additionally, using meta-regressions and meta-comparisons, we aim to investigate whether the strength of the association between these attitudes and the social perception of sexual violence is contingent upon attitudinal dimensions (gender-role expectations, hostile sexism, benevolent sexism, and rape myth acceptance), participant gender (women sample, men sample, and mixed sample), or the dyadic composition of the sexual violence incident (male perpetrator on female victim; female perpetrator on male victim; same-sex perpetrator and victim).

## Methodology

This meta-analysis was conducted according to the “Preferred Reporting Items for Systematic Reviews and Meta-Analyses” (PRISMA) guidelines (Page et al., 2021).

### Search Strategy

First, a comprehensive literature search was executed across two distinct electronic platforms (PsychArticles and Proquest) for articles published between January 1, 2010, and December 31, 2023, inclusively. Within these platforms, the following databases were used to perform the search: PsychINFO, PsychARTICLES, PsychTEST Proquest and ProQuest Dissertations & Theses Global. Second, a Google Scholar search was performed to retrieve published as well as unpublished work. Screening of the first 100 Google search results, organized by relevancy, was considered. Rayyan software was used to organize and facilitate the screening process (Ouzzani et al., 2016).

Specific keyword search was employed in conjunction with Boolean operators, including “AND,” and “OR,” facilitating the combination of search concepts and the inclusion of synonyms. Truncation symbols were also utilized to encompass all words commencing with the same sequence of letters. The search string was structured as follows: (“rape myth*” OR “rape supportive” OR “rape proclivity” OR “rape propensity” OR sexis* OR adversarial OR “sex* script*” OR dominan* OR “gender hierarchy” OR hostil* OR objectification OR dehumanization OR “sex* role*” OR “gender role*” OR masculin* OR feminin* OR hyperfeminin* OR hypermasculin*) AND (“sex* violen*” OR “sex* assault” OR “sex* aggress*” OR “sex* coerc*” OR “sex* offen*” OR rap*).

### Inclusion/Exclusion Criteria

Studies were included in the meta-analysis if they met the following criteria: (a) participants were at least 16 years of age; (b) materials depicting a sexually coercive interaction between two adults were provided (e.g., vignettes, auditory scenarios; videos); (c) at least one aspect of the social perception of sexual violence was rated after exposure to the provided material (victim blame attribution, perpetrator blame attribution, victim credibility rating, incident severity rating, victim experiencing trauma rating, victim experiencing pleasure rating, and sanction severity for the perpetrator); (d) measured at least one attitudes shaped by patriarchal socialization using an empirically validated scale; (e) provided original quantitative statistics on the relationship between attitudes shaped by patriarchal socialization and an aspect of social perception of sexual violence; and (f) provided sufficient data for effect size extraction. Studies were excluded if the sample included individuals convicted of sexual offences. All studies written in either English or French were considered. The first three authors of the article were actively engaged in determining eligibility. Each manuscript was assessed by two authors, with the first author assessing all manuscripts. Inter-rater agreement needed to be achieved to include or exclude a study, with any discrepancies resolved through consensus.

### Methodological Quality Assessment

Studies underwent a methodological appraisal to ensure the validity of the extracted data (Supplementary File 1). A seven-point scale was devised based on two authoritative sources: (i) the “Strengthening the Reporting of Observational Studies in Epidemiology” (STROBE) statement: guidelines for reporting observational studies (von Elm et al., 2008); and (ii) “The Quality Assessment Tool for Observational Cohort and Cross-Sectional Studies” (National Heart, Lung, and Blood Institute, 2017). Each of the 7 criteria was assigned a score of 0 (no) or 1 (yes), with a maximum total score of 7. Higher scores indicate greater methodological quality. Studies scoring between 0 and 2 were considered as low quality, those with scores between 3 and 5 as moderate quality, and those ranging from 6 to 7 as high quality. No studies were excluded from the meta-analysis for low quality. The first three authors conducted the quality assessment. Each manuscript was assessed by two authors, with any discrepancies resolved through consensus.

### Data Extraction

To be eligible for inclusion, studies were required to report any of the following statistical measures: Pearson correlation (*r*), odds ratios (*OR*s), means and standard deviations, Cohen's *d*, or Chi-square (*χ*^2^).

### Data Analysis

Data were collected and analyzed using Comprehensive Meta-Analysis Software version 4.0 (Biostat). Data are available at this link. All study results were transformed into Fisher’s correlation coefficient for analysis, with the results being converted back to the Pearson coefficient for ease of interpretation. Random effect meta-regression and meta-comparisons were performed to explore the influence of moderator variables on the association between attitudes shaped by patriarchal socialization and social perception of sexual violence. Variability was assessed using Cochran’s *Q* statistic and *I*^2^ statistic. Publication bias was evaluated through the Egger test and forest plots.

## Results

### Study Selection

**Figure 1.**
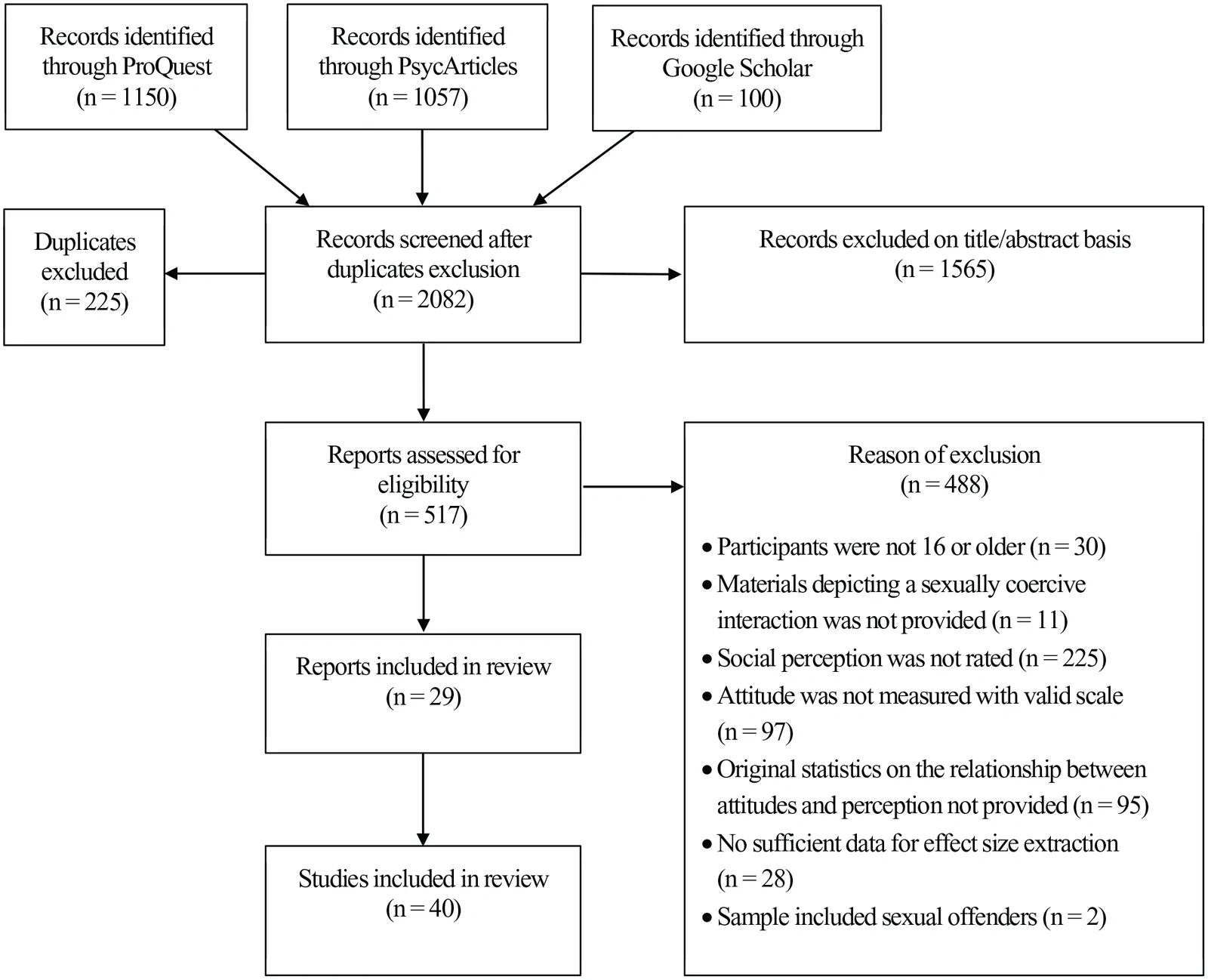
Selection process diagram.

A PRISMA flow diagram (Figure 1) displays the study selection process. Of the 2,082 records screened after duplicate exclusions, 1,565 were excluded after the title and abstract review. After a full-text examination of the remaining 517 reports, 29 met all inclusion criteria. Six of those reports included multiple studies. A total of 40 studies were included in the meta-analysis.

### Study Characteristics

Table 1 provides a synthesis of the characteristics of the included studies. Out of the 40 studies, 10 were unpublished dissertations. Studies were conducted across ten different countries, with the United States (*n* = 27), the United Kingdom (*n* = 4), and Hungary (*n* = 2) contributing multiple studies. The remaining seven studies were from Australia, Canada, Colombia, China, Italy, Spain, and Norway. The combined sample size across the 40 studies comprised 12,283 participants with a mean age of 27.20^2^ (17 to 82).

**Table 1.** Characteristics of the 40 Studies Included in the Meta-Analysis.

Author(s) (Year); Country	Sample Characteristics	Attitudinal Dimension(s):	Type of Material and Dyadic Interaction	Aspect of Social Perception and Correlation [95% CI]
1. Angelone et al. (2015); USA	*N* = 284; Men; *M*_age_ = 19.75	GRE: AWS- SF HS and BS: ASI	Vignettes man perp./woman vic.	GRE: Victim Blame: *r* = .29, [.18, .39] GRE: Perp. Blame: *r* = −.18, [−.29, −.07] GRE: Victim Credibility: *r* = −.30, [−.40 −.19] GRE: Severity: *r* = −.21, [−.32, −.10] GRE: Pleasure: *r* = .33, [.22, .43] GRE: Trauma: *r* = −.21, [−.32, −.10] GRE: Sanction: *r* = −.14, [−.25, −.02] HS: Victim Blame: *r* = .43, [.33, .52] HS: Perp. Blame: *r* = −.21, [−.32 −.10] HS: Victim Credibility: *r* = −.42, [−.51, −.32] HS: Severity: *r* = −.30, [−.40 −.19] HS: Pleasure: *r* = .34, [.23, .44] HS: Trauma: *r* = −.23, [−.34, −.18] HS: Sanction: *r* = −.18, [−.29 −.07] BS: Victim Blame: *r* = .07, [−.05, .19] BS: Perp. Blame: *r* = −.05, [−.17, .07] BS: Victim Credibility: *r* = −.05, [−.17, .07] BS: Severity: *r* = .02, [−.10, .14] BS: Pleasure: *r* = .05, [−.07, .17] BS: Trauma: *r* = −.02, [−.14, .10] BS: Sanction: *r* = .05, [−.07, .17]
2. Angelone et al. (2018); USA	*N* = 626; Mixed sample; *M*_age_ = 19.5	RMA: IRMA HS and BS: ASI	Vignettes man perp. / woman vic.	RMA: Victim Blame: *r* = .44, [.40, .50] RMA: Perp. Blame: *r* = −.31, [−.37, −.23] RMA: Victim Credibility: *r* = −.44, [−.50, .38] RMA: Severity: *r* = −.42, [−.48, −.35] HS: Victim Blame: *r* = .32, [.25, .39] HS: Perp. Blame: *r* = −.16, [−.24 −.08] HS: Victim Credibility: *r* = −.30, [−.37, −.23] HS: Severity: *r* = −.28, [−.35 −.21] BS: Victim Blame: *r* = .011, [.04, .19] BS: Perp. Blame: *r* = −.05, [−.13, .03] BS: Victim Credibility: *r* = −.27, [−.34, −.19] BS: Severity: *r* = .02, [−.06, .10]
3. Ayala et al. (2018); USA	*N* = 221; Women; 18–58 y/o; *M*_age_ = 21.38	RMA: IRMA-SF	Vignettes man perp. / woman vic. woman perp. / man vic. man perp. / man vic. woman perp./ woman vic.	RMA: Victim Blame: *r* = .35, [.23, .46] RMA: Perp. Blame: *r* = −.13, [−.26, −.00] * Only pooled together bivariate correlations were available in the article.
4. Basow and Minieri (2011); USA	*N* = 185; Mixed sample; 17–22 y/o; *M*_age_ = 19.07	RMA: IRMA-SF	Vignettes man perp. / woman vic.	RMA: Victim Blame: *r* = .51, [.40, .61] RMA: Perp. Blame: *r* = −.21, [−.34, −.07]
5. Bendixen et al. (2014); Norvegia	*N* = 455; Mixed sample; *M*_age_ = 41.01	RMA: IRMA-SF	Vignettes man perp. / woman vic.	RMA: Victim Blame: *r* = .67, [.62, .72]
6. Bevens et al. (2018, std1); USA	*N* = 189; Women; 18+ y/o; *M*_age_ = 18.88	RMA: U-IRMA	Scene from a film man perp. / woman vic.	RMA: Victim Blame: *r* = .60, [.50, .68]
7. Bevens et al. (2018, std2); UK	*N* =103; Women; 18+ y/o	RMA: ARVS	Vignette man perp. / woman vic.	RMA: Victim Blame: *r* = .66, [.54, .76]
8. Binion (2021); USA	*N* = 1181; Mixed sample; 18–75 y/o; *M*_age_ = 35.92	RMA: IRMA-SF	Vignettes man perp. / woman vic. woman perp. / man vic. man perp./ man vic. woman perp./ woman vic.	W/W RMA: Victim Blame: *r* = .68, [.65, .71] W/M RMA: Victim Blame: *r* = .79, [.77, .81] M/M RMA: Victim Blame: *r* = .77, [.75, .79] M/W RMA: Victim Blame: *r* = .77, [.75, .79] M/M RMA: Victim Blame: *r* = .81, [.79, .83] M/M RMA: Victim Blame: *r* = .77, [.75, .79]
9. Boppana (2021); USA	*N* = 265; Mixed sample; 18–25 y/o; *M*_age_ = 20.11	RMA: MRMS; IRMA-SF	Vignettes man perp. / woman vic. woman perp. / man vic.	M/W RMA: Victim Blame: *r* = .74, [.68, .79] W/M RMA: Victim Blame: *r* = .87, [.84, .90]
10. Chahal et al. (2022); USA	*N* = 846; Mixed sample; 18–46 y/o	Usexism: GBSI RMA : U-IRMA; SAMDSR	Vignettes man perp. / woman vic. woman perp./ man vic. man perp./ man vic. woman perp./ woman vic.	RMA: Victim Blame: *r* = .45, [.40, .50] RMA: Perp. Blame: *r* = −.42, [−.47, −.36] RMA: Sanction: *r* = −.30, [−.36, −.24] Usexism: Victim Blame: *r* = .33, [.27, .39] Usexism: Perp. Blame: *r* = −.31, [−.37, −.25] Usexism: Sanction: *r* = −.21, [−.27, −.15] * Only pooled together results were available in the article.
11. Clippert-Treadwell (2014); USA	*N* = 149; Mixed sample; 19–50 y/o; *M*_age_ = 26.00	RMA: IRMA-SF	Vignettes man perp. / woman vic.	RMA: Victim Blame: *r* = .58, [.46, .68] RMA: Perp. Blame: *r* = −.57, [−.67, −.45]
12. Dawtry et al. (2019, Std1); UK	*N* = 255; Mixed sample; *M*_age_ = 36.89	RMA: IRMA-SF	Vignette man perp. / woman vic.	RMA: Victim Blame: *r* = .77, [.72, .82]
13. Dawtry et al. (2019, Std2); UK	*N* = 405; Mixed sample; *M*_age_ = 37.69	RMA: IRMA-SF	Vignette man perp. / woman vic.	RMA: Victim Blame: *r* = .76, [.72, .80]
14. Durán and Rodríguez-Domínguez (2020); Spain	*N* = 301; Mixed sample; 18–27 y/o; *M*_age_ = 20.50	HS and BS: ASI	Vignettes man perp. / woman vic.	HS: Victim Blame: *r* = .53, [.44, .61] HS: Perp. Blame: *r* = −.32, [−.42 −.22] HS: Severity: *r* = −.35, [−.44 −.61] BS: Victim Blame: *r* = .51, [.42, .59] BS: Perp. Blame: *r* = −.30, [−.40, −.19] BS: Severity: *r* = −.39, [−.48, .29]
15. Hammond et al. (2011); USA	*N* = 172; Mixed sample; 18+ y/o	RMA: RMAS	Vignette man perp. / woman vic.	RMA: Victim Blame: *r* = .44, [.31, .55] RMA: Perp. Blame: *r* = −.29, [−42, −.14]
16. Hargitay (2017); USA	*N* = 106; Mixed sample; 18−64+ y/o	RMA: AMMSA	Vignettes man perp. / woman vic.	RMA: Victim Blame: *r* = .48, [.31, .61] RMA: Perp. Blame: *r* = −.54, [−.66, −.39]
17. Hogge and Wang (2022, Phase 2); USA	*N* = 275; Mixed sample; 18–29 y/o; *M*_age_ = 18.75	RMA: MRMS-R; U-IRMA	Vignettes woman perp. / man vic. man perp. / man vic.	woman perp. / man vic. RMA(IRMA) : Vic.Blame: *r* = .28, [.17, .39] RMA(MRMS) : Vic.Blame: *r* = .39, [.29, .49] man perp. / man vic. RMA(IRMA) : Vic.Blame: *r* = .38, [.27, .48] RMA(MRMS) : Vic. Blame: *r* = .45, [.35, .54]
18. Katz et al. (2018); USA	*N* = 172; Women; 17–24 y/o; *M*_age_ = 18.89	HS and BS: ASI	Vignette man perp. / woman vic.	HS: Victim Blame: *r* = .43, [.30, .55] BS: Victim Blame: *r* = .37, [.23, .49]
19. Klement (2017, Std 1A); USA	*N* = 97; Mixed sample; *M*_age_ = 22.43	RMA: U-IRMA	Vignettes man perp. / woman vic.	RMA: Victim Blame: *r* = .51, [.35, .64] RMA: Perp. Blame: *r* = −.28, [−.45 −.09] RMA: Victim Cred. *r* = −.67, [−.77, −.54] RMA: Severity: *r* = −.46, [−.60 −.29] RMA: Pleasure: *r* = .20, [.00, .38] RMA: Trauma: *r* = −.44, [−.59, −.26] RMA: Sanction: *r* = −.15, [−.34 −.05]
20. Klement (2017, Std 1B); USA	*N* = 112; Mixed sample; *M_a_*_ge_ = 34.32	RMA: U-IRMA	Vignettes man perp. / woman vic.	RMA: Victim Blame: *r* = .72, [.62, .80] RMA: Perp. Blame: *r* = −.49, [−.62 −.34] RMA: Victim Cred. *r* = −.65, [−.75, −.53] RMA: Severity: *r* = −.40, [−.55 −.23] RMA: Pleasure: *r* = .60, [.47, .71] RMA: Trauma: *r* = −.48, [−.61, −.32] RMA: Sanction: *r* = −.08, [−.26 .11]
21. Klement (2017, Std 2); USA	*N* = 84; Mixed sample; *M*_age_= 22.43	RMA: U-IRMA	Vignettes man perp. / woman vic.	RMA: Victim Blame: *r* = .79, [.69, .86] RMA: Perp. Blame: *r* = −.40, [−.57 −.20] RMA: Victim Cred. *r* = −.72, [−.81, −.60] RMA: Severity: *r* = −.57, [−.70 −.41] RMA: Pleasure: *r* = .40, [.20, .57] RMA: Trauma: *r* = −.40, [−.60, −.25] RMA: Sanction: *r* = −.33, [−.51 −.12]
22. Klement (2017, Std 3A); USA	*N* = 98; Mixed sample; *M*_age_ = = 20.25	RMA: MRMS	Vignettes woman perp./ man vic.	RMA: Victim Blame: *r* = .56, [.41, .68] RMA: Perp. Blame: *r* = −.21, [−.39 −.01] RMA: Victim Cred. *r* = −.36, [−.52, −.17] RMA: Severity: *r* = −.40, [−.55 −.22] RMA: Pleasure: *r* = .54, [.38, .67] RMA: Trauma: *r* = −.38, [−.54, −.20] RMA: Sanction: *r* = −.16, [−.35 .04]
23. Klement (2017, Std 3B); USA	*N* =104; Mixed sample; *M*_age_ = 33.62	RMA: MRMS	Vignettes woman perp. / man vic.	RMA: Victim Blame: *r* =.70, [.59, .79] RMA: Perp. Blame: *r* = −.11, [−.30 −.08] RMA: Victim Cred. *r* = −.45, [−.59, −.28] RMA: Severity: *r* = −.17, [−.35 .02] RMA: Pleasure: *r* = .62, [.49, .73] RMA: Trauma: *r* = −.34, [−.50, −.16] RMA: Sanction: *r* = .11, [−.08 .30]
24. Klement (2017, Std 4); USA	*N* = 116; Mixed sample; *M*_age_ = 19.91	RMA: MRMS	Vignettes woman perp. / man vic.	RMA: Victim Blame: *r* = .57, [.43, .68] RMA: Perp. Blame: *r* = −.40, [−.54 −.24] RMA: Victim Cred. *r* = −.64, [−.74, −.52] RMA: Severity: *r* = −.62, [−.72 −.49] RMA: Pleasure: *r* = .54, [.40, .66] RMA: Trauma: *r* = −.61, [−.71, −.48] RMA: Sanction: *r* = −.29, [−.45 −.11]
25. Klement et al. (2019, Std 1); USA	*N* = 97; Mixed sample; 18–45 y/o; *M*_age_ = 22.43	RMA: U-IRMA	Vignettes man perp. / woman vic.	RMA: Victim Blame: *r* = .51, [.35, .64] RMA: Perp. Blame: *r* = −.28, [−.45 −.09] RMA: Victim Cred. *r* = −.67, [−.77, −.54] RMA – Severity: *r* = −.46, [−.60 −.29] RMA: Pleasure: *r* = .20, [.00, .38] RMA: Trauma: *r* = −.44, [−.59, −.26] RMA: Sanction: *r* = −.15, [−.34, .05]
26. Klement et al. (2019, Std 2); USA	*N* = 84; Mixed sample; 19–40 y/o; *M*_age_ = 22.43	RMA: U-IRMA	Vignettes man perp. / woman vic.	RMA: Victim Blame: *r* = .79, [.69, .86] RMA: Perp. Blame: *r* = −.40, [−.57 −.20] RMA: Victim Cred. *r* = −.72, [−.81, −.60] RMA: Severity: *r* = −.57, [−.70 −.41] RMA: : Pleasure: *r* = .40, [.20, .57] RMA: Trauma: *r* = −.44, [−.60, −.25] RMA: Sanction: *r* = −.33, [−.51 −.12]
27. Klement et al. (2019, Std3); USA	*N* = 98; Mixed sample; 18–28 y/o; *M*_age_= 20.25	RMA: MRMS	Vignettes man perp. / woman vic.	RMA: Victim Blame: *r* = .56, [.41, .68] RMA – Victim Cred. *r* = −.36, [−.52, −.17] RMA: Severity: *r* = −.52, [−.65 −.36] RMA: Pleasure: *r* =.54, [.38, .67] RMA: Trauma: *r* = −.38, [−.54, −.20] RMA: Sanction: *r* = −.16, [−.35 −.04]
28. Klement et al. (2019, Std4); USA	*N* = 116; Mixed sample; 18–26 y/o; *M*_age_ = 19.91	RMA: MRMS	Vignettes man perp. / woman vic.	RMA: Victim Blame: *r* = .57, [.43, .68] RMA: Perp. Blame: *r* = −.40, [−.54 −.24] RMA: Victim Cred. *r* = −.64, [−.74, −.52] RMA: Severity: *r* = −.62, [−.72 −.49] RMA: Pleasure: *r* =. 54, [.40, .66] RMA: Trauma: *r*= −.61, [−.71, −.48] RMA: Sanction: *r* = −.29, [−.45 −.11]
29. Li and Zheng (2022); China	*N* = 1011; Mixed sample; *M*_age_ = 28.37	RMA: IRMA	Vignettes man perp. / woman vic.	RMA: Victim Blame: *r* = .38, [.33, .43] RMA: Perp. Blame: *r* = −.30, [−.36 −.24]
30. McDaniel and Rodriguez (2021); USA	*N* = 218; Men; 18–24 y/o	GRE: MRNI RMA: AMMSA	Vignette man perp. / woman vic.	GRE: Victim Blame: *r* = .39, [.27, .50] GRE: Perp. Blame: *r* = −.31, [−.43 −.19] RMA: Victim Blame: *r* = .50, [.39, .59]
31. Milesi et al. (2020); Italy	*N* = 553; Mixed sample; *M*_age_ = 23.18	RMA: AMMSA	Vignette man perp. / woman vic.	RMA: Victim Blame: *r* = .64, [.59, .69]
32. Nyúl et al. (2018, Std1); Hungary	*N* = 870; Mixed sample; *M*_age_ = 37.52	RMA: U-IRMA	Real case man perp. / woman vic.	RMA: Severity: *r* = −.19, [−.25 −.13] RMA: Sanction: *r* = −.27, [−.33 −.22]
33. Nyúl et al. (2018, Std2); Hungary	*N* = 105; Mixed sample; *M*_age_ = 21.78	RMA: U-IRMA	Real case man perp. / woman vic.	RMA: Sanction: *r* = −.44, [−.58 −.27]
34. O’Connor et al. (2021); USA	*N* = 512; Mixed sample; 18–67 y/o; *M*_age_ = 27.24	Usexism: MSS	News clippings man perp. / woman vic. (single victim) man perp. / woman vic. (multiple victims)	Single victim RMA: Sanction: *r* = .08, [−.01 .17] Multiple victims RMA: Sanction: *r*= −.36, [−.43 −.28]
35. Oesterle et al. (2023); USA	*N* = 610; Men; 18–22 y/o; *M*_age_= 18.28	RMA: IRMA-SF	Vignettes man perp. / woman vic.	RMA: Severity: *r* = −.35, [−.41 −.27]
36. Persson and Dhingra (2022, Std2); UK	*N* = 252; Mixed sample; 18–82 y/o; *M*_age_ = 35.31	RMA: AMMSA HS and BS: ASI	Vignettes man perp. / woman vic.	RMA: Victim Blame: *r* = .47, [.37, .56] RMA: Perp. Blame: *r* = −.33, [−.43 −.22] HS: Victim Blame: *r* = .47, [.37, .56] HS: Perp. Blame: *r* = −.30, [−.41 −.18] BS: Victim Blame: *r* = .29, [.17, .40] BS: Perp. Blame: *r* = −.21, [−.33 −.09]
37. Saldarriaga et al. (2023, Stsd2); Colombia	*N* = 180; Mixed sample, *M*_age_ = 27.96	RMA: AMMSA	Vignette man perp. / woman vic.	RMA: Victim Blame: *r* = .63, [.53, .71] RMA: Perp. Blame: *r* = −.24, [−.37 −.10]
38. Starfelt et al. (2015); Australia	*N* = 210; Mixed sample, 18–25 y/o; *M*_age_ = 20.00	GRE: SRQ RMA: IRMA-SF	Vignette man perp. / woman vic.	GRE: Victim Blame: *r* = .28, [.15, .40] GRE: Perp. Blame: *r* = −.10, [−.23; .04] RMA: Victim Blame: *r* = .46, [.35, .56] RMA: Perp. Blame: *r* = −.33, [−.45 −.20]
39. Wilson et al. (2021); USA	*N* = 397; Women, 17–49 y/o; *M*_age_= 19.02	RMA: U-IRMA	Vignettes man perp. / woman vic.	RMA: Victim Blame: *r* = .48, [.40, .55]
40. Zidenberg et al. (2021); Canada	*N* = 168; Mixed sample; *M*_age_ = 25.15	RMA: U-IRMA	Mock police report man perp. / woman vic.	Mixed sample : RMA: Victim Blame: *r* = .63, [.53, .72] RMA: Perp. Blame: *r* = −.41, [−.53 −.28] Women participants: RMA: Victim Blame: *r* = .58, [.42, .70] Men participants: RMA: Victim Blame: *r* = .57, [.46, .67]

*Note*. AMMSA = Acceptance of Modern Myths about Sexual Aggression (Gerger et al., 2007); ASI = Ambivalent Sexism Inventory (Glick & Fiske, 1996); ARVS = Attitude Toward Rape Victims Scale (Ward, 1988); AWS-SF = Attitudes Toward Woman Scale- Short Form (Spence et al., 1978); BS = Benevolent sexism; GBSI = Gender Blind Sexism Inventory (Stoll et al., 2017); GRE = Gender role expectations; HS = Hostile sexism; IRMA = Illinois Rape Myth Acceptance Scale (Payne et al., 1999); IRMA-SF = Illinois Rape Myth Acceptance Scale- Short Form (Payne et al., 1999); U-IRMA = Updated Illinois Rape Myth Acceptance Scale (McMahon & Farmer, 2011); MRMS = Male Rape Myth Scale (Melanson, 1999); MRMS-R = Male Rape Myth Scale—revised (Hogge & Wang, 2022); MRNI = Male Role Norms Inventory (Levant et al., 2010); MSS = Modern Sexism Scale (Swim et al., 1995); RMA = Rape myth acceptance; RMAS = Rape Myth Acceptance Scale (Burt, 1980); SAMDSR = Sexual Assault Myths Downplaying the Severity of Rape (Stoll et al., 2017); SRQ = Social Roles Questionnaire (Baber & Tucker, 2006).

To facilitate moderation analyses, attitudes were categorized according to attitudinal dimension. As most studies focusing on sexism differentiated between hostile sexism and benevolent sexism, data related to sexism were analyzed according to these two distinct dimensions.^3^ Thus, four attitudinal dimensions were considered for moderation analysis: gender role expectations, hostile sexism, benevolent sexism, and rape myth acceptance.

A substantial number of studies measured more than one attitudinal dimension (*n* = 9; 22.5%). Most studies (*n* = 26; 65.0%) also explored various aspects of social perception. Seven quantifiable aspects of the social perception of a sexual violence incident were identified: blame attribution to the victim; blame attribution to the perpetrator; victim credibility assessment; incident severity assessment; perception of the victim experiencing pleasure; acknowledgment of the victim’s trauma; and sanction severity for the perpetrator. Consult Table 1 for details regarding the measured attitudinal dimensions and aspects of social perception for each study. All combinations between an attitudinal dimension and one of the seven aspects of social perception were individually entered and analyzed. As a result, 172 independent combinations were obtained and analyzed.

### Main Results

A total of 172 independent results were gathered to determine the strength of the association between attitudes shaped by patriarchal socialization (pooled-together attitudinal dimensions) and the social perception of a sexual violence incident. Table 2 presents the detailed number of samples, effect sizes, and confidence intervals for each of the seven social perception aspects. Effect directions for the seven aspects were standardized before running the main analysis. A random-effect meta-analysis rendered a statistically significant overall effect size of *r* = .425 (95% CI = .385; .463; *Z* = 18.688, *p* < .001). Statistically significant heterogeneity was found between studies (*Q* = 4797.912, *p* < .001, *I*^2^ = 96,436). Risk of bias due to study quality was assessed and was not significant (*Q* = 3,33, *p* = .189). No publication bias was detected (*t* = 1.539, *p* = .125).

**Table 2.** Number of Samples, Effect Sizes, and Confidence Intervals on the Relation Between Attitudes Stemming from Patriarchal Socialization and the Seven Aspects of Social Perception.

Social Perception Aspects	Sample Number	*r*	95% CI
Blame attribution to the victim	58	.558	.499 to .612
Blame attribution to the perpetrator	33	−.288	−.330 to −.244
Victim credibility rating	16	−.492	−.574 to −.400
Incident severity rating	20	−.363	−.438 to −.284
Perception of the victim experiencing pleasure	13	.417	.311 to .512
Acknowledgment of the victim’s trauma	13	−.390	−.486 to −.283
Sanction severity for the perpetrator	19	−.191	−.260 to −.121
Overall effect of attitudes on social reaction^a^	172	.425	.385 to .463

a To compute overall social reaction, effect directions were standardized.

To delve deeper into between studies heterogeneity, a random effect meta-regression tested the variance explained by attitudinal dimensions (gender-role expectations, hostile sexism, benevolent sexism, and rape myth acceptance), participant gender (women sample, men sample, and mixed sample), and the dyadic composition of the incident (male perpetrator on female victim; female perpetrator on male victim; same-sex perpetrator and victim). The model^4^ significantly explained 45% of the total between-study variance (*Q* = 60.44, *p* < .0001). Both attitudinal dimensions (*Q* = 24.32; *p* < .0001) and dyadic composition (*Q* = 8.73, *p* < .01) emerged as significant moderators, while participant gender did not yield a significant contribution to the model (*Q* = 3.40, *p* = .183).

Significant moderators were further explored using mixed-effects meta-comparisons. Results highlight the significant contribution of all attitudinal dimensions to the social perception of sexual violence (refer to Table 3 for specific details). Substantial differences in effect sizes among the various attitudinal dimensions were identified (*Q* = 77.469, *p* ≤ .001). The effect size associated with rape myth acceptance was significantly larger than that of all other attitudinal dimensions. Additionally, hostile sexism exhibited a statistically equivalent effect size to gender role expectation and a significantly greater effect size compared to benevolent sexism.

**Table 3.** Effect Sizes and Z-Scores According to Attitudinal Dimensions.

Attitudinal Dimensions	Sample number	*r*	95% CI	*Z*	*p <*
Rape myth acceptance	117	.499	.455 to .541	18.675	.001
Hostile sexism	17	.328	.279 to .376	12.242	.001
Benevolent sexism	17	.165	.087 to .242	4.109	.001
Gender role expectations	11	.250	.199 to .300	9.310	.001

Significant distinctions were also observed in the effect sizes among the three dyadic compositions (*Q* = 18.199, *p* < .001). The effect size documented for incidents involving same-gender perpetrators and victims (*n* = 6; *r* = .675; 95% CI = .566, .761) was significantly greater than those involving female perpetrators on male victims (*n* = 38; *r* = .468; 95% CI = .392; .538) or male perpetrators on female victims (*n* = 116; *r* = .400; 95% CI = .355; .444).

## Discussion

In this meta-analysis, our primary objective was to quantitatively assess the strength of the relationship between attitudes shaped by patriarchal socialization and the perception of sexual violence. Additionally, through meta-regressions and meta-comparisons, we aimed to investigate whether the strength of this association was contingent upon attitudinal dimensions, participant gender, or the dyadic composition of the incident.

Results from a combined sample of 12,283 participants across 40 studies from 10 different countries revealed a significant relationship between attitudes shaped by patriarchal socialization and the perception of sexual violence. Specifically, the overall effect size of *r* = .425 is considered a strong effect size (Hemphill, 2003). These attitudes were strongly correlated with increased blame attribution to the victim, reduced credibility afforded to the victim, lower ratings of both incident severity and acknowledgment of the victim’s trauma, as well as higher ratings of the victim deriving pleasure from the incident. Meanwhile, they showed a moderate correlation with blame attribution to the perpetrator and a weak correlation to sanction severity for the perpetrator. It thus appears that attitudes shaped by patriarchal socialization most strongly influence the negative perception of victims and the trivialization of sexual violence, whereas its impact on the perception of perpetrators or our inclination to punish them is comparatively more subtle.

Meta-regressions highlighted the influence of attitudinal dimensions as moderators. First, rape myth acceptance emerged as a major contributor to tolerant response toward sexual violence. The considerable volume of research on rape myth acceptance, along with the consistency of findings across these studies, suggests that these beliefs should remain a focal point for education, awareness campaigns, and prevention programs. These programs should be evidence-based, inclusive of both men and women, and developed within a framework of progressive education, characterized by repetition and increasing content complexity over time (Orchowski et al., 2018).

Second, both hostile sexism and gender role expectations were, respectively, found to be strongly and moderately associated with the social perception of sexual violence. Benevolent sexism also emerged as a significant contributor, although its effect size is weaker than the other dimensions. These findings suggest that, beyond rape myth acceptance, upholding traditional gender roles and stereotypical behavioral expectations for both men and women, significantly influences how incidents of sexual violence and victims are perceived. Results also support the already established tendency to consider sexism as a bi-dimensional concept.

Meta-regressions also highlighted that the dyadic composition of sexual violence incidents has an impact on how it is perceived. Findings indicate a significant increase in effect size when the incidents involved same-gender perpetrators and victims. This outcome potentially points to the over-stigmatization of sexual and gender minorities victims (Edwards et al., 2023) and underscores the role of gendered social norms, heteronormativity and sexual scripts in shaping our societal understanding and perception of sexual violence. Results also align with studies emphasizing the disparities experienced by individuals diverging from heterocisnormative social norms (Girshick, 2002; Martin-Storey et al., 2018; Mortimer et al., 2019; Stemple & Meyer, 2014).

Finally, participant gender did not emerge as a significant moderator in the model. These results do not imply that there are no differences between men and women in terms of their level of endorsement of these attitudes. A strong body of evidence demonstrates that men do endorse these attitudes significantly more than women (Baril et al., 2025; Hockett et al., 2016; Suarez, & Gadalla, 2010). What these results suggest is that the influence of these attitudes on the perception of sexual violence is the same for both men and women. Thus, regardless of their gender, the more one adheres to these attitudes, the more they present biased interpretations of events related to sexual violence. It thus appears imperative that awareness and education efforts be directed toward the entire population, irrespective of gender.

In conjunction with sociocultural models, our findings underscore the significance of sexism, gender-role expectations and rape myth acceptance in the social perception of sexual violence (Table 4 presents a synthesis of the critical findings). Specifically, these attitudes negatively influence perceptions of victim responsibility and credibility as well as the awareness of sexual violence consequences, three dimensions that may influence the social support provided to a victim following disclosure (Ahrens et al., 2007; Franklin & Garza, 2021). Whether disclosure happens through formal or informal sources, negative reactions—such as victim blaming or minimizing the events or their consequences—have serious ramifications for victims, such as increasing the expression of post-traumatic symptoms and reducing chances of recovery (Jacques-Tiura et al., 2010; Ullman, 2023). It seems imperative to foster individuals’ ability to receive and respond appropriately to victims’ disclosures as well as continue to promote awareness and provide education and programs that can effectively reduce tolerant attitudes toward sexual violence (Tibbels & Benbouriche, 2024).

**Table 4.** Table of Critical Findings.

• When considered collectively, attitudes shaped by patriarchal socialization (sexism, gender role expectations and rape myth acceptance) are strongly correlated to the social perception of sexual violence. Additionally, when assessed as distinct moderators, gender role expectations, hostile sexism, benevolent sexism, and rape myth acceptance all emerged as significant contributors to the social perception of sexual violence. • Attitudes shaped by patriarchal socialization were strongly correlated with increased blame attribution to the victim, reduced credibility afforded to the victim, lower ratings of both incident severity and acknowledgment of the victim's trauma, as well as higher ratings of the victim deriving pleasure from the incident. These attitudes also showed a moderate correlation with blame attribution to the perpetrator and a weak correlation with sanction severity for the perpetrator. • The relationship between attitudes shaped by patriarchal socialization and the social perception of sexual violence was significantly stronger when incidents involved same-gender perpetrators and victims compared to those involving perpetrators and victims of opposite genders, regardless of whether the perpetrator was a man or woman. • Meta-regression revealed that participants’ gender did not significantly moderate the relationship between attitudes shaped by patriarchal socialization and the social perception of sexual violence.

### Limitations

This meta-analysis has limitations. Some limitations are related to the characteristics of the included studies. The depiction of individuals involved in the sexual violence incidents described in the vignettes was highly heterogeneous, typically featuring a female victim, a male perpetrator, and the presumption of heterosexuality. Out of the 165 available results, only six addressed same-sex dyads, five of which involved male-on-male dyads. Little to no attention was given to other marginalized sociodemographic characteristics or identities, such as people of color (1 study), gender minorities (0 studies), individuals of lower socioeconomic status (1 study), individuals with disabilities (0 studies), or individuals with larger body sizes (1 study). These characteristics are known to elicit heightened negative social reactions toward victims of sexual violence. (Ullman, 2023). Similarly, the lack of significant differences observed between participant genders should also be interpreted with caution, as most results (133/164) were derived from convenience samples, for which no gender-specific results were reported. Only five results were based on exclusively female samples, while 26 were available for exclusively male samples. Non-significant results could thus be attributable to insufficient statistical power. Many studies assessed multiple attitudes or aspects of social perception, leading to the same samples contributing to multiple independent results. Finally, results of this research are based on participants' perceptions of an analogous sexual violence situation, which may differ from their perceptions of a real-life situation.

Some limitations are inherent to the meta-analytic process. First, this meta-analysis is based on 40 distinct studies that examined four different attitudes and their relations to seven aspects of the social perceptions of sexual violence. While efforts were made to ensure consistency across studies—such as applying rigorous inclusion criteria and conducting inter-rater assessments—an inherent limitation of meta-analyses is the potential variability in how different studies conceptualize, operationalize, and measure the same constructs. This factor should be considered when interpreting the findings. Also, our keyword search string did not include terms related to victimization or perpetration, which may have caused studies to be excluded from the results.

### Future Directions

These findings have important practical implications (Table 5 synthesizes implications for practice, policy, and research). Results highlight several key targets for education and prevention. While efforts to combat rape myth acceptance should remain central to these initiatives, our findings suggest that reducing social tolerance toward sexual violence also requires addressing gender-based stereotypes and gender inequity. Unlike rape myths, which are typically challenged later in life, efforts to address gendered expectations and biases can begin in early childhood and be reinforced and adapted throughout various developmental stages. Additionally, it is crucial to raise awareness to gender biases and foster the ability to appropriately receive and respond to disclosures of sexual violence, particularly among professionals who may encounter such disclosures, to improve support for victims and strengthen overall prevention strategies.

**Table 5.** Table of Implications for Practice, Policy, and Research.

Implications for Practice • Rape myths are to remain a central target of awareness campaigns, education, and prevention programs. • Awareness and education initiatives should address gendered expectations and power dynamics, be grounded in a framework of progressive education, and be directed toward the entire population. • Educational efforts should foster the ability to appropriately receive and respond to disclosures of sexual violence, particularly among professionals likely to encounter such disclosures, in order to enhance support for victims and strengthen overall prevention strategies. Implications for Policy • Reducing social tolerance toward sexual violence should be integrated into broader initiatives that target gender biases and power dynamics. • Policies should be inclusive of gender and sexual minorities and account for the over-stigmatization of social groups that experience systemic discrimination. • Efforts should be made for policies to challenge the underlying structures that perpetuate gender biases and power dynamics. Meaningful changes require broader societal restructuring that addresses the distribution of resources, power, and status across individuals and groups with diverse social identities. Implications for Research • Findings underscore gaps in knowledge due to gender-specific and heteronormative bias in current sexual violence research, highlighting the need for including women, and individuals from sexual and gender diversity in research protocols, using inclusive questionnaires and materials and moving away from stereotypical depiction of rape and sexual violence. • Findings underscore the significance of unequal power dynamics embedded in socialization processes and how these dynamics shape the social perception of sexual violence. From this perspective, future research should strongly consider adopting an intersectional lens to more comprehensively capture the complexity of sexual violence. • Researchers are encouraged to include comprehensive and detailed descriptions of the materials used in sexual violence research, either within the methods section or in supplementary materials. Providing such information is essential for enhancing the precision, transparency, and replicability of future studies in this field.

These findings also have implications for research. Future sexual violence research must address the clear heteronormative and cisnormative biases that shape current knowledge. Considering that women, as well as sexual and gender minorities, are particularly affected by both gendered norms and sexual violence and that this meta-analysis positions gender hierarchy and gender-role expectations as factors influencing social perception of sexual violence, inclusivity in research becomes imperative. Researchers must move away from stereotypical representations of sexual violence and work to include more diverse participants and perspectives in both research design and materials. Researchers should also be more intentional in providing comprehensive and detailed descriptions of the materials used in sexual violence research, either within the methods section or as supplementary files. Such details are essential for improving the accuracy and depth of future research in this field.

This manuscript emphasizes the significance of unequal power dynamics in our socialization and how these dynamics influence the social perception of sexual violence. From this perspective, future research on sexual violence should strongly consider adopting an intersectional lens. Accordingly, the issue of gender hierarchy should also be considered in interaction with other social statuses known to face systemic discrimination, such as Black and racialized individuals, people with disabilities, members of the LGBTQ+ community, neurodivergent individuals, those with low income or limited education, and people of different ages. Belonging to one of these groups, coupled with sexual victimization, could intensify negative perceptions and behaviors toward survivors of sexual violence. Adopting an intersectional lens will ensure that future research more comprehensively addresses the complexities of sexual violence.

## Conclusion

This meta-analysis expands the current literature by quantifying the extent of the relationship between various attitudes shaped by patriarchal socialization and the perception of sexual violence, its victims, and perpetrators. It provides several insights that will help prioritize prevention efforts more effectively. Considering the findings, reducing social tolerance toward sexual violence should not only target rape myths acceptance but also be integrated into broader initiatives that target gender biases and promote gender-equal relationships. It is imperative to consider addressing gender role expectations as well as gender hierarchy from an early age, as a precursor to sexual education and sexual health promotion efforts while also persisting in raising awareness and implementing educational programs that can effectively influence rape myths and other tolerant attitudes toward sexual violence. In that sense, efforts should aim to challenge the underlying structures that perpetuate gender biases and power dynamics. Ultimately, meaningful attitude and behavior changes require larger societal restructuring that addresses the distribution of resources, power, and status among individuals and groups of diverse social identities.

## Supplemental Material

sj-docx-1-tva-10.1177_15248380251343190 – Supplemental material for The Effect of Gender Role Expectations, Sexism, and Rape Myth Acceptance on the Social Perception of Sexual Violence: A Meta-Analysis Supplemental material, sj-docx-1-tva-10.1177_15248380251343190 for The Effect of Gender Role Expectations, Sexism, and Rape Myth Acceptance on the Social Perception of Sexual Violence: A Meta-Analysis by Dominique Trottier, Valérie Laviolette, Irza Tuzi and Massil Benbouriche in Trauma, Violence, & Abuse

## Notes

1. In the context of this meta-analysis, the term “social perception of sexual violence” refers to the subjective evaluation of a sexual violence situation and the people involved in the incident. We believe that this term best encompasses the variations in conceptualizations across included studies (e.g., perception of rape, moral judgment on rape, social perception of sexual assault, judgment of sexual assault, recognition of sexual assault, perceived victim/perpetrator blame rape attribution, blame attribution, ascribed culpability, judgment of the accuser, and attribution of responsibility).

2. Based on the 35 studies that provided means.

3. Undifferentiated sexism samples (*n* = 5) were excluded from the regression model to prevent multicollinearity.

4. The model is based on the 164 independent results with complete data for all moderators.
