# Supplementary material for: The Effect of Gender Role Expectations, Sexism, and Rape Myth Acceptance on the Social Perception of Sexual Violence: A Meta-Analysis
Source: Trauma Violence Abuse. 2025 Jun 21;27(4):1270–86. doi: 10.1177/15248380251343190 (PMC13578662; doi:10.1177/15248380251343190)
Supplement: Supplementary file 1 [file sj-docx-1-tva-10.1177_15248380251343190.docx]

Appendix A. *Quality scoring for studies included in the meta-analysis*

|  | Research question | Defined sample | Sample size  justification | Eligibility criteria | Validated measure  (attitude) | Identified sources of bias or limitations | Published study | Total score  /7 |
| --- | --- | --- | --- | --- | --- | --- | --- | --- |
| 1. Angelone et al. (2015) | YES | YES | NO | NO | YES | YES | YES | 5/7 |
| 2. Angelone et al. (2018) | YES | YES | NO | NO | YES | YES | YES | 5/7 |
| 3. Ayala et al. (2018) | YES | YES | YES | NO | YES | YES | YES | 6/7 |
| 4. Basow & Minieri (2011) | YES | YES | NO | NO | YES | YES | YES | 5/7 |
| 5. Bendixen et al. (2014) | YES | YES | YES | NO | YES | YES | YES | 6/7 |
| 6. Bevens et al. (2018, Study 1) | YES | YES | YES | NO | YES | YES | YES | 6/7 |
| 7. Bevens et al. (2018, Study 2) | YES | NO | NO | NO | YES | YES | YES | 4/7 |
| 8. Binion (2021) | YES | YES | NO | YES | YES | YES | NO | 5/7 |
| 9. Boppana (2021) | YES | YES | YES | YES | YES | YES | NO | 6/7 |
| 10. Chahal et al. (2022) | YES | YES | YES | YES | YES | YES | YES | 7/7 |
| 11. Clippert-Treadwell (2014) | NO | YES | YES | YES | YES | YES | NO | 5/7 |
| 12. Dawtry et al. (2019, Study 1) | YES | YES | YES | NO | YES | YES | YES | 6/7 |
| 13. Dawtry et al. (2019, Study 2) | YES | YES | YES | NO | YES | YES | YES | 6/7 |
| 14. Duran & Rodriguez-Dominguez (2020) | YES | YES | NO | NO | YES | YES | YES | 5/7 |
| 15. Hammond et al. (2011) | YES | YES | NO | NO | YES | YES | YES | 5/7 |
| 16. Hargitay (2017) | NO | YES | NO | YES | YES | YES | NO | 4/7 |
| 17. Hogge & Wang (2022, Phase 2) | YES | YES | NO | NO | YES | YES | YES | 5/7 |
| 18. Katz et al. (2017) | YES | YES | NO | NO | YES | YES | YES | 5/7 |
| 19. Klement (2017, Study 1A) | YES | YES | NO | NO | YES | YES | NO | 4/7 |
| 20. Klement (2017, Study 1B) | YES | YES | NO | NO | YES | YES | NO | 4/7 |
| 21. Klement (2017, Study 2) | NO | YES | NO | NO | YES | YES | NO | 3/7 |
| 22. Klement (2017, Study 3A) | YES | YES | NO | NO | YES | YES | NO | 4/7 |
| 23. Klement (2017, Study 3B) | YES | YES | NO | NO | YES | YES | NO | 4/7 |
| 24. Klement (2017, Study 4) | NO | YES | NO | NO | YES | YES | NO | 3/7 |
| 25. Klement (2019, Study 1) | YES | YES | NO | NO | YES | YES | YES | 5/7 |
| 26. Klement (2019, Study 2) | YES | YES | NO | NO | YES | YES | YES | 5/7 |
| 27. Klement (2019, Study 3) | YES | YES | YES | NO | YES | YES | YES | 6/7 |
| 28. Klement (2019, Study 4) | YES | YES | YES | NO | YES | YES | YES | 6/7 |
| 29. Li & Zheng (2022) | YES | YES | YES | YES | YES | YES | YES | 7/7 |
| 30. McDaniel & Rodriguez (2021) | YES | YES | YES | NO | YES | YES | YES | 6/7 |
| 31. Milesi et al. (2019) | YES | YES | NO | YES | YES | YES | YES | 6/7 |
| 32. Nyul et al. (2018, Study 1) | YES | YES | NO | NO | YES | YES | YES | 5/7 |
| 33. Nyul et al. (2018, Study 2) | YES | YES | NO | NO | YES | YES | YES | 5/7 |
| 34. O’Connor et al. (2021) | YES | YES | NO | NO | YES | YES | YES | 5/7 |
| 35. Oesterle et al. (2023) | YES | YES | YES | NO | YES | YES | YES | 6/7 |
| 36. Persson & Dhingra (2022, Study 2) | YES | YES | YES | NO | YES | YES | YES | 6/7 |
| 37. Saldarriga et al. (2023, Study 2) | NO | YES | YES | NO | YES | YES | YES | 5/7 |
| 38. Starfelt et al. (2015) | YES | YES | NO | NO | YES | YES | YES | 5/7 |
| 39. Wilson et al. (2021) | YES | YES | NO | NO | YES | YES | YES | 5/7 |
| 40. Zidenberg et al. (2021) | YES | YES | NO | NO | YES | YES | YES | 5/7 |

**Criterias addapted from :**

Von Elm E, Altman D.G., Egger, M. et al. (2007). Strenghtening the reporting of observational studies in epidemiology (STROBE)

statement: guidelines for reporting observational studies. Annals of internal medicine; 147, 573 – 577. <https://doi.org/10.7326/0003-4819-147-8-200710160-00010>

National Heart, Lung, and Blood Institute. (2017). Quality Assessment Tool for Observational Cohort and Cross-Sectional Studies.

<https://www.nhlbi.nih.gov/health-topics/study-quality-assessment-tools>)

**Criterias selected according to recommendations by:**

Sanderson, S, Tatt, I. D. & Higgins, J. P. T. (2007) Tools for assessing quality and susceptibility to bias in observational studies in

epidemiology: a systematic review and annotated bibliography. International Journal of Epidemiology; 36, 666–676.

<https://doi.org/10.1093/ije/dym018>
